# MRI of Syrian hamsters reveals SARS-CoV-2 variant-specific neuroinflammation

**DOI:** 10.1128/spectrum.03567-25

**Published:** 2026-02-03

**Authors:** Yisi Tang, Xiaohui Wei, Kai Gao, Bowen Guan, Jiangning Liu, Xudong Shi

**Affiliations:** 1 CAMS & PUMC, NHC Key Laboratory of Human Disease Comparative Medicine, Institute of Laboratory Animal Science, National Human Diseases Animal Model Resource Center, National Center of Technology Innovation for Animal Model, State Key Laboratory of Respiratory Health and Multimorbidity, Beijing, China; Uniwersytet Medyczny w Bialymstoku, Bialystok, Poland

**Keywords:** SARS-CoV-2 variants, neuroimaging, hamster, histopathology, neuroinflammation

## Abstract

SARS-CoV-2 infection causes neurological manifestations in 30%–40% of patients, with reinfection exacerbating sequelae risks. Elucidating neuroinvasion mechanisms requires optimized animal models overcoming limitations of existing systems. This study establishes the Syrian hamster as a neuroanatomically optimized model to investigate SARS-CoV-2 variant-specific neuropathogenesis. Syrian hamsters subjected to primary infection and reinfection with WH-09 and Omicron subvariants (XBB.1, BA.1, or BF.7) were investigated using multimodal MRI and histopathology. We demonstrate that SARS-CoV-2 variants exhibit strikingly differential neuroinvasive capacities following primary infection and reinfection. Notably, immune-evasive variants like XBB.1 and WH-09 induced significant, unresolved damage in olfactory-limbic regions, correlating with clinical symptoms such as hyposmia. BF.7 and BA.1 exhibited minimal neurostructural alterations in both primary and reinfection models. These variant-specific neuropathological profiles, precisely mapped via MRI-histopathology correlation, underscore the model’s utility in delineating the neurological threats posed by evolving variants.

**IMPORTANCE:**

This study demonstrates that Syrian hamsters offer advantages for modeling SARS-CoV-2 neuropathogenesis. WH-09 and XBB.1’s persistent olfactory and hippocampus damage, which was quantified through integrated MRI and histopathology, underscores the neurological threat posed by immune-evasive variants. Conversely, Omicron’s attenuated phenotype provides a blueprint for virulence-modifying interventions. This work establishes multimodal imaging in hamsters as an effective strategy for evaluating emerging viral variants.

## INTRODUCTION

Since its emergence in late 2019, SARS-CoV-2 and the resulting COVID-19 disease have presented a major global public health challenge. Beyond respiratory symptoms, substantial clinical evidence confirms the significant neuroinvasive potential of SARS-CoV-2. Approximately 30%–40% of patients develop neurological manifestations, including acute symptoms such as hyposmia, headache, impaired consciousness, and even encephalitis, as well as long-term sequelae like anxiety, depression, and cognitive impairment (1–3). Of particular note, reinfection may further exacerbate the risk of neurological sequelae. Elucidating the mechanisms by which SARS-CoV-2 affects the central nervous system (CNS) is crucial for developing effective neuroprotective strategies (4, 5). The animal models serve as indispensable tools, providing a critical platform for investigating viral neuroinvasion mechanisms, pathological progression, and potential interventions (6, 7). By simulating infection with different viral strains (e.g., Delta, Omicron), the dynamic impact of the virus on brain structure and function will be revealed, overcoming the challenge in clinical studies of obtaining pre- and post-infection comparative data.

The precise route of SARS-CoV-2 invasion into the CNS remains incompletely elucidated (8, 9). Animal models have provided critical insights into this process. Transgenic mouse models expressing the human angiotensin-converting enzyme 2 (hACE2) receptor successfully recapitulate viral entry into the brain via the olfactory nerve pathway (10, 11). Notably, significant differences exist in neuroinvasive capacity among viral variants. In K18-hACE2 mouse models, the Delta variant exhibits greater neuroinvasiveness than Omicron, resulting in more severe encephalitis and higher mortality (12, 13). Mechanistic studies reveal that Delta achieves significantly higher replication efficiency in neural cells compared to Omicron, potentially attributable to differential spike protein cleavage efficiency. In contrast, Omicron predominantly exerts indirect effects on brain tissue by activating neuroglial cells and mediating immune-inflammatory responses (14).

Magnetic resonance imaging (MRI), with its high resolution and multi-parametric capabilities, has emerged as an ideal strategy for assessing structural and functional neurological changes in animals after infection with SARS-CoV-2 variants. In patients infected with SARS-CoV-2, elevated T2 signal intensity was observed in the olfactory bulb, hippocampus, and frontal cortex by post-infection day 7, suggesting inflammatory edema. Diffusion tensor imaging (DTI) demonstrated reduced fractional anisotropy (FA) values in the corpus callosum, indicating myelin structural damage (15, 16). In Non-Human Primates infected with the Delta variant (B.1.617.2), MRI revealed marked reductions in gray matter volume and compromised white matter integrity (17). Histopathological examination confirmed microglial activation, astrogliosis, and neuronal apoptosis in these regions. These findings align with human autopsy studies showing significant neuroinflammation and neuronal loss in the brain tissues of Delta variant fatalities (18).

**Fig 1:**
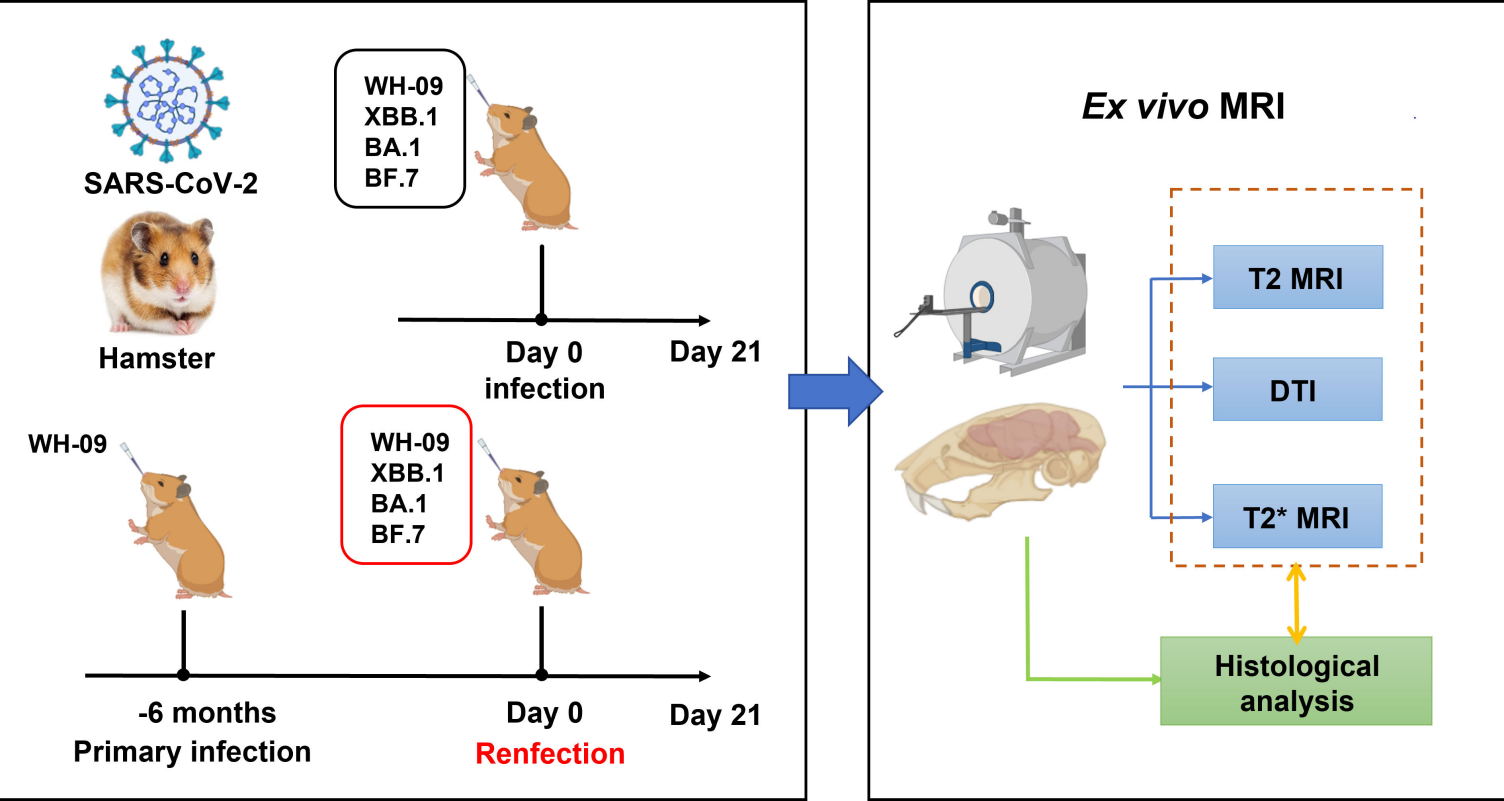
Study design. Syrian hamsters were intranasally inoculated with the four SARS-CoV-2 strain**s** (WH-09 strain, Omicron BA.1, BF.7, and XBB.1) at an equal dose of 1 × 10^5^ TCID_50_ per hamster. Furthermore, Syrian hamsters initially infected with the WH-09 strain were rechallenged with the four virus strains 6 months later. After 21 days post-infection, e*x vivo* MRI and histological study were performed.

Syrian hamster (*Mesocricetus auratus*) is a robust model for SARS-CoV-2 pathogenesis, recapitulating key features of human infection, including robust viral replication in the upper respiratory tract, transient lung pathology, and neuroinvasion via the olfactory route (19–21). This study will employ hamster models infected with distinct SARS-CoV-2 variants (WH-09, XBB.1, BA.1, BF.7) to characterize brain structural alterations via MRI (Fig. 1). Meanwhile, MRI manifestations in hamster models following heterologous rechallenge with distinct viral variants at 6 months post-primary infection with WH-09 were also investigated. Histopathological validation was integral to confirm and interpret the imaging findings. We aim to establish *ex vivo* MRI as a sensitive tool for characterizing variant-specific neurovirulence in preclinical models.

## RESULTS

### Hamster brain atlas and MRI template

**Fig 2:**
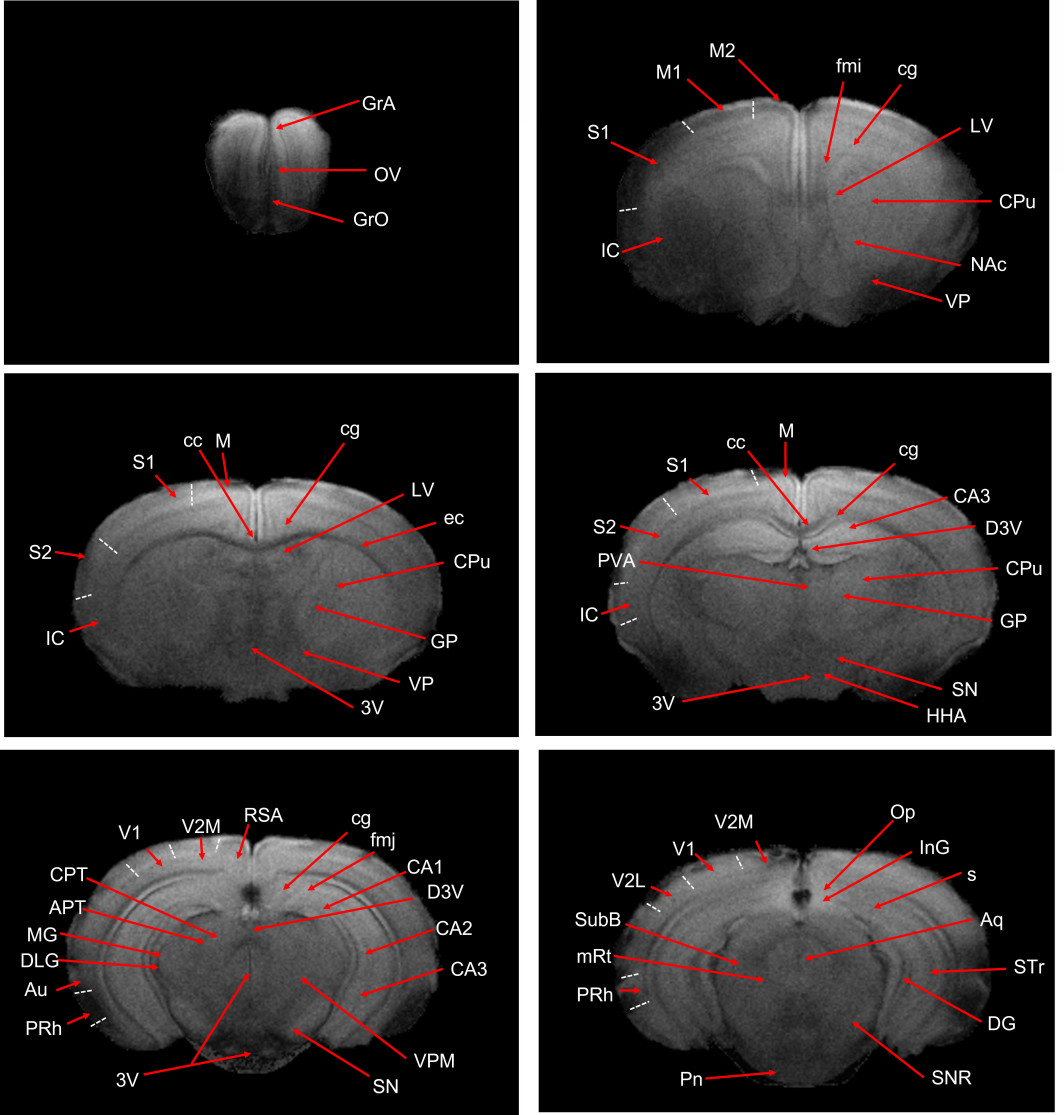
Six representative T2*-weighted coronal images with structural annotations (abbreviations correspond to Table S1).

**Fig 3:**
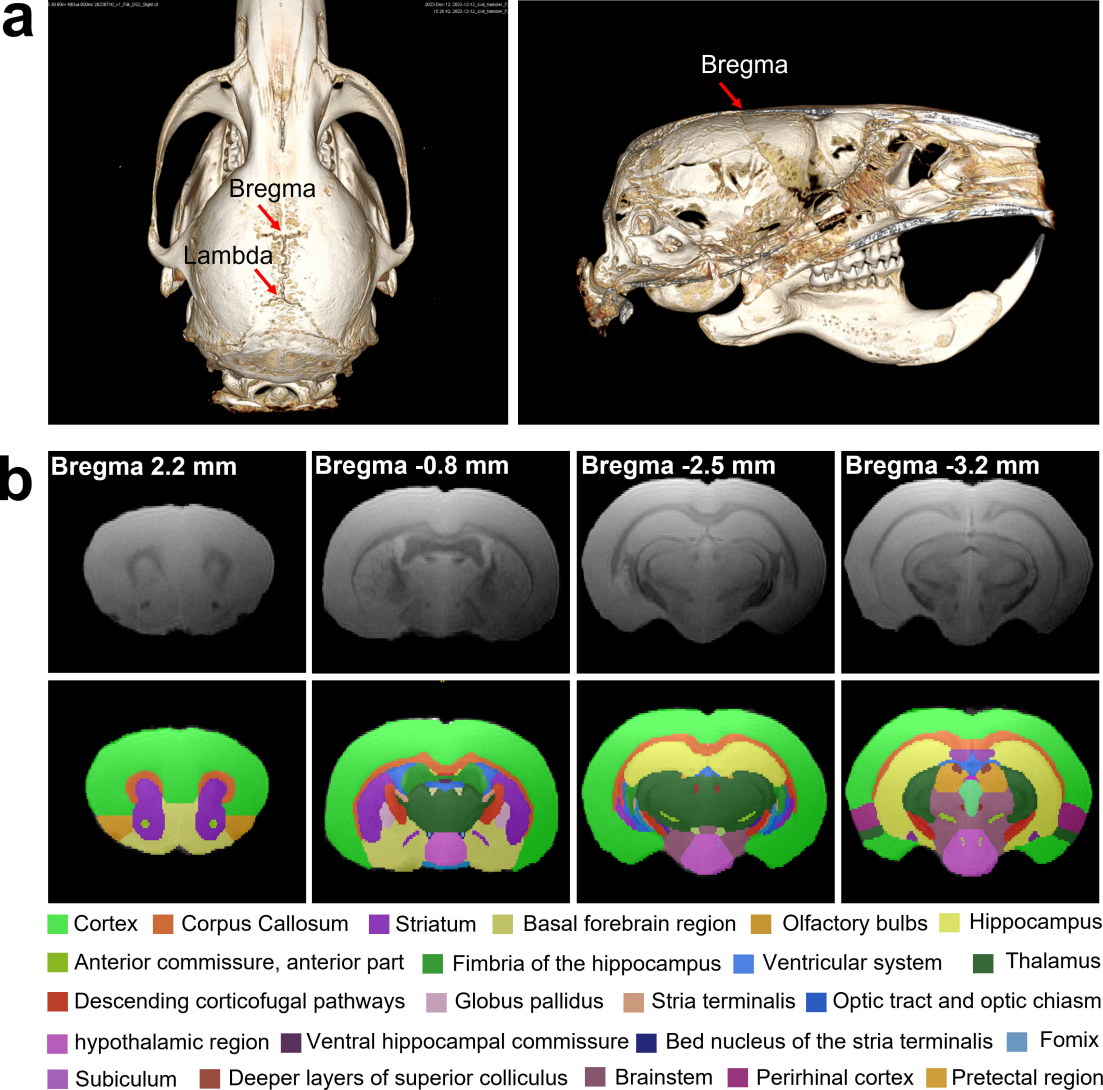
(**a**) CT images of the head of a hamster with the brain. (**b**) The labeled MRI template of the hamster brain with the axial plane.

Due to slightly higher water content in brain tissue, hamsters may exhibit lower gray-white matter contrast (22) at identical magnetic field strengths (e.g., 7.0 T MRI). Therefore, we utilized T2*-weighted structural MRI to delineate and annotate major brain structures (Fig. 2) referencing the histochemical brain atlas of Syrian hamsters (23). The MRI template (Fig. 3) was constructed for annotation of the hamster brain using T2* images of eight healthy hamsters (6–8 weeks old). This atlas provides an anatomical reference framework for future MRI-based research on neurological and psychiatric disease models of hamsters. In comparison to C57BL/6 mice, hamsters possess a larger brain volume and enhanced development of hypothalamic nuclei such as the suprachiasmatic nucleus, which confers enhanced capacity for circadian and seasonal behavioral regulation. Moreover, the olfactory bulbs in hamsters are proportionally larger (occupying ~5% of total brain volume [Fig. S2] versus 2%–3% in mice), reflecting their olfactory-dependent behavioral ecology. This neuroanatomical feature further highlights their utility as models for respiratory infectious disease research.

### T2 and T2* MRI of the brain in hamsters infected with SARS-CoV-2

The inability to accommodate high-field small-animal MRI systems within biosafety level 3 (BSL-3) laboratories necessitated *ex vivo* MRI scanning of SARS-CoV-2-infected hamster brain tissues. To comparatively assess inter-strain pathogenicity, Syrian hamsters (*n* = 6 per group, with equal numbers of males and females) received intranasal inoculations of WH-09, BA.1, BF.7, or XBB.1 followed by terminal anesthesia at 21 dpi. Furthermore, Syrian hamsters initially infected with the WH-09 strain were rechallenged with four variants after a 6-month convalescence period, with tissue collection at 21 days post-reinfection (dpr).

**Fig 4:**
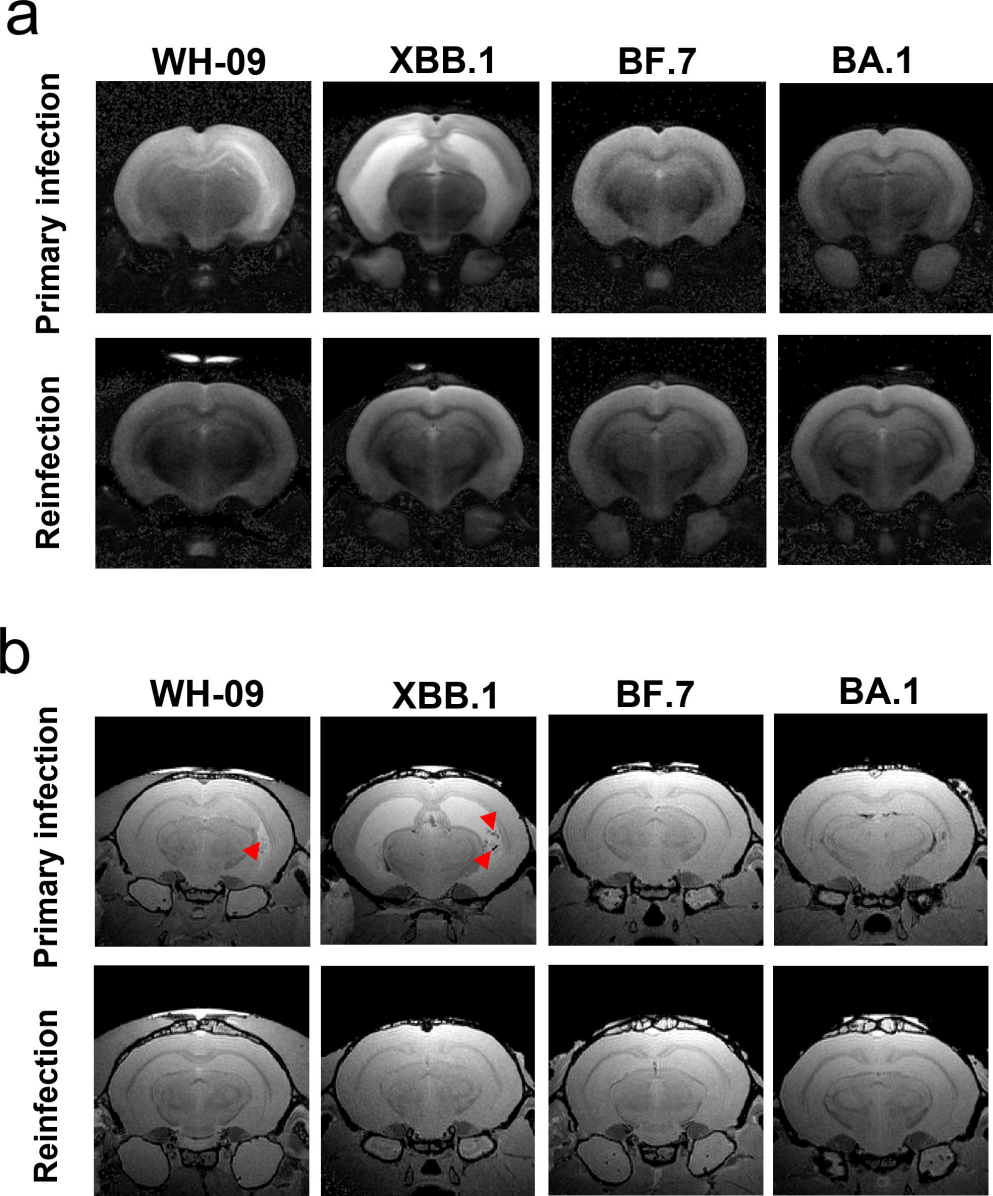
T2 (**a**) and T2* (**b**) brain MRI images of a hamster with primary infection and reinfection by different SARS-CoV-2 strains. The red triangles indicate hemorrhagic spots.

T2-weighted imaging revealed significant hyperintense signals (Fig. 4a) in the parahippocampal gyrus of hamsters at 21 days post-primary infection with WH-09 and XBB variants, indicating edema or inflammatory changes. This phenomenon may be attributed to SARS-CoV-2 infection, which activates microglia and astrocytes, triggering the release of proinflammatory cytokines (e.g., IL-6, TNF-α) and consequent neuroinflammation. Compared to WH-09, the XBB.1 variant exhibits enhanced neuroinvasiveness due to its superior immune evasion capability, facilitating blood-brain barrier penetration and persistent viral accumulation. This leads to chronic neurodegenerative alterations, manifested as extensive bilateral hippocampal hyperintensity on T2-weighted images (Fig. 4a). Notably, the hippocampal regions affected by viral invasion were significantly smaller in WH-09-infected hamsters than in those infected with XBB.1.

Concurrently, virus-mediated vascular endothelial injury and increased blood-brain barrier permeability resulted in microhemorrhages (50–200 μm in diameter) detectable via T2*-weighted imaging in WH-09- and XBB-infected hamster (Fig. 4b). These microhemorrhages were predominantly distributed in subcortical white matter and thalamic regions, aligning with neuroimaging findings in clinical cases of COVID-19. After secondary infection with WH-09 and XBB in hamsters initially infected with WH-09, no significant signal abnormalities were observed in T2- and T2*-weighted images of olfactory bulbs and hippocampus. The degree of neural damage was markedly lower than during primary infection, indicating that immune protection generated by initial infection can suppress neuroinflammatory responses to secondary infection. Consistent with clinical reports, Omicron subvariants (BA.1/BF.7) induced primarily localized metabolic disturbances in neural pathology—a conclusion validated in the hamster model. No significant imaging abnormalities were observed on either T2- or T2*-weighted sequences following BA.1/BF.7 infection.

### DTI of the brain in a hamster infected with SARS-CoV-2

**Fig 5:**
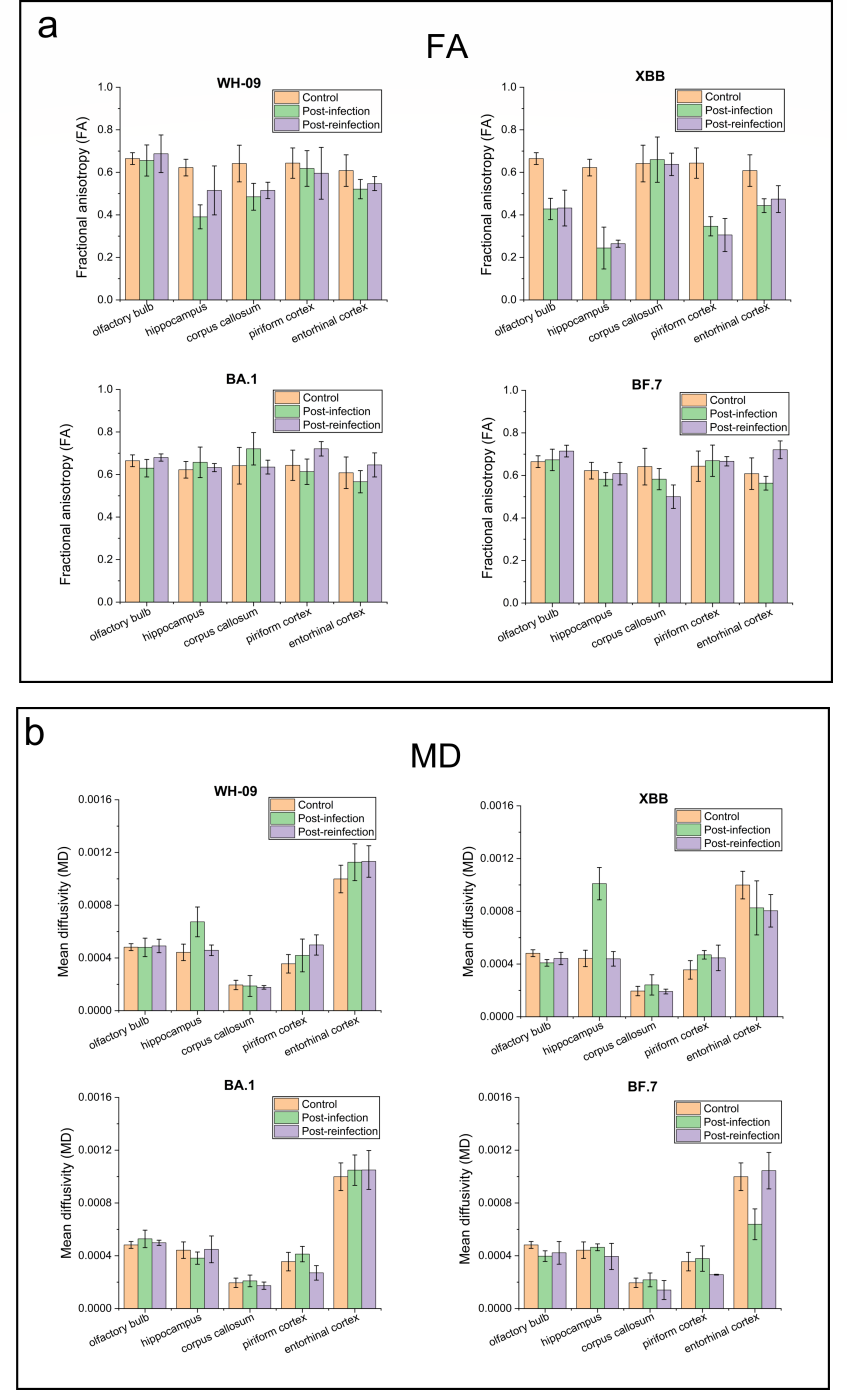
FA (**a**) and MD (**b**) of brain regions (olfactory bulb, hippocampus, corpus callosum, piriform cortex, entorhinal cortex) in hamsters with primary infection and reinfection by different SARS-CoV-2 strains.

DTI analysis (Fig. 5) revealed that at 21 days post-primary WH-09 infection, Syrian hamster models exhibited significantly reduced FA in the corpus callosum (−24.3%, *P* = 0.016) and hippocampus (−37.3%, *P* = 0.037), indicating axonal damage. Concurrently, the hippocampus demonstrated substantial increases in radial diffusivity (RD, +93.7%, *P* = 0.038) and mean diffusivity (MD, +52.2%, *P* = 0.08), suggesting demyelination associated with direct viral invasion of oligodendrocytes. The change of MD in the hippocampus of hamsters infected with WH-09 showed a statistical trend. These changes were consistent with blood-brain barrier disruption leading to intravascular fluid extravasation into the extracellular space, correlating with prominent inflammatory manifestations in hippocampal regions on T2-weighted imaging. Following secondary infection with WH-09, FA reductions in the hippocampus and corpus callosum were attenuated (−17.2% and −19.6%, respectively), with no significant alterations in MD or RD values.

Notably, primary XBB.1 infection elicited more pronounced neuroinvasiveness than WH-09, as evidenced by DTI metrics: hippocampal FA decreased markedly in both primary (−60.8%, *P* = 6.3E-6) and secondary XBB.1 infection (−57.5%, *P* = 0.0022) groups. This phenomenon is attributed to XBB.1’s potent immune evasion capacity, facilitating persistent viral presence in the brain, which may induce chronic inflammatory demyelination. Primary XBB.1 infection induced dramatic hippocampal RD and MD elevations (+128.5% and +205.6%), whereas secondary infection caused negligible changes. Distinct from WH-09 infection, the olfactory bulb displayed significant DTI alterations in XBB.1-infected hamsters, with FA reductions of −35.7% (primary, *P* = 0.039) and −34.9% (secondary, *P* = 0.0010). Concomitant FA decreases in the entorhinal and piriform cortices implicated olfactory pathway disruption, indicating XBB.1-induced hyposmia in hamsters.

In contrast, BF.7/BA.1 showed no significant FA or MD changes in major tracts, including olfactory bulb, corpus callosum, and hippocampus, consistent with their lower neuroinvasive potential.

### Histological features of the brain in hamsters infected with SARS-CoV-2

**Fig 6:**
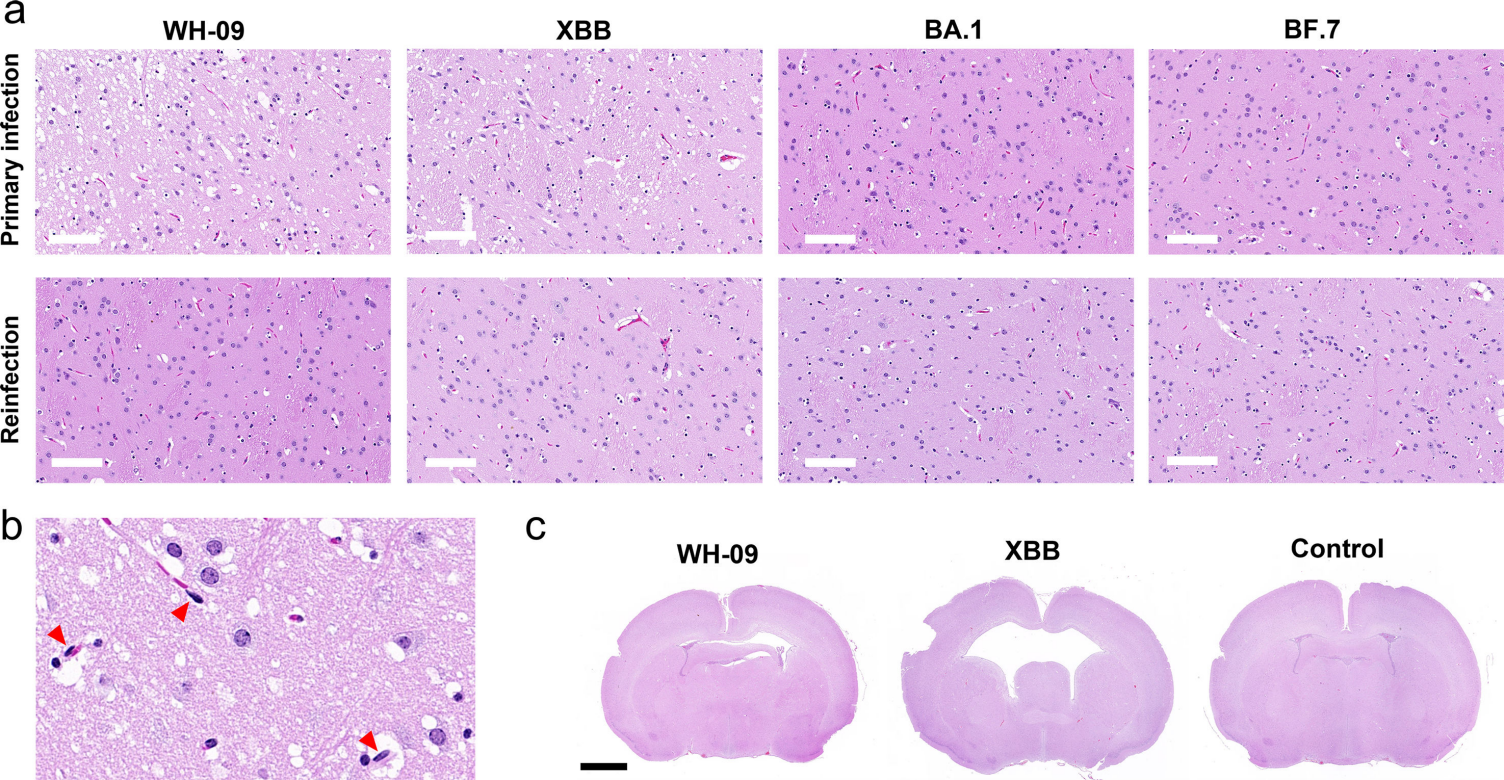
Histopathological images of hippocampus (**a**) and whole brain (**c**) in hamster with primary infection and reinfection by different SARS-CoV-2 strains, the scale bar is 100 μm and 2 mm, respectively. (**b**) The enlarged hippocampus of WH-09, primarily infected hamster, red triangles indicating rod-shaped microglia. The scale bar is 20 μm.

Histopathological analysis (Fig. 6) of hematoxylin and eosin (H&E)-stained sections revealed extensive neuronal damage in the hippocampus of Syrian hamsters at 21 days post-primary WH-09 infection. Gliotic edema and perivascular space widening were observed, indicating WH-09 induced cerebrovascular inflammation and blood-brain barrier disruption. Rod-shaped microglial cells (Fig. 6b) were observed in the hippocampal region, an indicator of viral infection causing pathological elongation or proliferation of microglia. In contrast, primary XBB.1 infection produced severe ventriculomegaly with marked cerebral ventricular dilation. Hippocampal gliovascular proliferation and white matter vacuolation were significantly more pronounced in XBB.1-infected hamsters compared to WH-09 cohorts, suggesting accelerated demyelination processes. The heightened neuroinvasiveness of XBB.1 was consistent with human autopsy evidence demonstrating persistent viral presence in neural tissue.

Secondary infection (either WH-09 or XBB) following primary WH-09 exposure resulted in substantially attenuated neuronal and glial damage compared to primary infection alone. Notably, BF.7/BA.1 exhibited minimal histopathological alterations in both primary and secondary infection models. No significant neuronal degeneration was detected, and inflammatory responses remained subdued, aligning with their reduced neuroinvasive potential.

## DISCUSSION

The clinical evidence indicates that SARS-CoV-2 infection can manifest with discernible neurological alterations, including abnormalities detectable on brain MRI. Reported findings in COVID-19 patients encompass inflammatory changes within the brain parenchyma or meninges, potentially reflecting virally triggered immune responses, cerebrovascular complications such as infarction or hemorrhage in rare instances (24, 25). These neuroimaging findings suggest that SARS-CoV-2 can induce neurological damage either through direct neuroinvasion or via immune-mediated mechanisms, leading to neuronal injury and inflammation. Notably, olfactory and gustatory dysfunction are highly prevalent neuropsychiatric features during the subacute phase, with studies reporting olfactory dysfunction (hyposmia or anosmia) in up to 86% and taste dysfunction (hypogeusia) in 86% of patients (26, 27). The hACE2 transgenic model developed by Qin demonstrated the presence of viral antigens in the olfactory bulb and brainstem regions post-infection, indicating trans-synaptic viral propagation along olfactory pathways, ultimately reaching brainstem respiratory centers (28). This discovery provides direct evidence explaining the neural mechanisms underlying hyposmia and respiratory failure in patients.

The Syrian hamster model overcomes mouse limitations through natural ACE2 receptor compatibility, enabling authentic infection (29). It uniquely demonstrates early SARS-CoV-2 neuroinvasion via peripheral nerves before viremia and models long-term neurological sequelae, like neuroinflammation and proteinopathies, providing a critical system for studying COVID-19’s acute and lasting effects on the nervous system (30). This study establishes the Syrian hamster as a neuroanatomically optimized model for SARS-CoV-2 neuropathogenesis research, leveraging its proportionally enlarged olfactory bulbs (~5% vs. 2%–3% brain volume in mice (31) and hypertrophied hypothalamic nuclei to overcome limitations of rodent models. Through T2*-weighted MRI protocols that resolved inherent gray-white matter contrast challenges at 7.0 T, we generated the annotated brain atlas for Syrian hamster—a critical foundation for respiratory virus studies. Crucially, multimodal imaging revealed divergent neuropathogenic mechanisms between the WH-09 and XBB.1 variants. WH-09 and XBB.1 primarily induced significant neuroinflammation, evidenced by T2 hyperintensity in the parahippocampal gyrus, which indicates edema from microglial/astrocyte activation and IL-6/TNF-α release. Furthermore, T2* imaging detected associated microhemorrhages (50–200 μm) in the subcortical white matter and thalamus. XBB.1 exhibited superior neuroinvasiveness with 2.3-fold larger bilateral hippocampal lesions. That was attributed to immune evasion facilitated by BBB penetration and persistent viral replication. Histopathological validation revealed severe ventriculomegaly and gliovascular proliferation in the XBB.1 variant, differing markedly from the neuronal pyknosis and perivascular edema seen in WH-09.

DTI metrics further quantified variant-dependent white matter vulnerability: WH-09 primary infection caused axonal damage (corpus callosum FA↓24.3%, hippocampus FA↓37.3%) and demyelination (hippocampal RD↑93.7%), attenuated post-secondary infection (FA↓17.2%–19.6%), indicating partial immune protection. Conversely, XBB.1 drove persistent hippocampal degeneration (FA↓57.5%–60.8%; RD↑128.5% in primary infection) and severe olfactory pathway disruption (olfactory bulb FA↓35.7%, entorhinal/piriform cortex FA↓), providing the microstructural evidence for XBB.1-induced hyposmia that correlated with histopathological vacuolation. These changes aligned with human autopsy evidence of persistent viral nucleocapsid in neural tissue, substantiating trans-olfactory spread as the dominant neuroinvasion route (32). Notably, Omicron BA.1/BF.7 showed no significant T2/T2*/DTI abnormalities and minimal histopathology (neuronal loss), confirming clinically reported neurotropism attenuation (12, 33).

The convergence of imaging and histopathology illuminates three principles. It reveals that immune evasion capacity dictates neurovirulence progression. For instance, XBB.1’s antibody resistance enables chronic demyelination and ventriculomegaly. Furthermore, clinical symptoms like hyposmia (from a 35.7% decrease in bulb FA) and cognitive deficits (from a 205.6% increase in hippocampal RD) stem from olfactory-limbic vulnerability. Lastly, secondary infection outcomes diverge by variant, as immune memory attenuates WH-09 damage but fails against XBB.1’s persistence. While limited to acute/subacute phases and male cohorts, this approach enables future work to map long-term sequelae and evaluate olfactory-targeted interventions against immune-evasive variants. Most critically, XBB’s unresolved neural damage underscores ongoing neurological threats from evolving SARS-CoV-2 lineages, whereas attenuation of BA.1 and BF.7 offers mechanistic insights for virulence modulation.

The inability to accommodate high-field small-animal MRI systems within biosafety level 3 (BSL-3) laboratories necessitated *ex vivo* MRI scanning of SARS-CoV-2-infected hamster brain tissues. Furthermore, it was not possible to conduct sequential MRI observations of neuroinflammatory lesion progression in the same hamster at different time points after viral infection. Thus, this study precluded the characterization of dynamic neuropathological progression and potential long-term outcomes. Although *in vivo* MRI scanning of live hamsters was not performed in this study, *ex vivo* MRI scans of brain tissues collected immediately following euthanasia and tissue fixation still effectively revealed differences in neuroinflammatory responses induced by different SARS-CoV-2 variants.

In this study, the hamsters subjected to primary infection and reinfection with WH-09 and Omicron subvariants (XBB.1, BA.1, or BF.7) were investigated. The variant-dependent structural and microstructural alterations were observed specifically within the olfactory bulb, piriform cortex, and connected limbic regions (hippocampus, amygdala). Combining MRI images with detailed histopathology provides superior spatial correlation. The MRI imaging corresponding to the histopathological images of inflammation, edema, and neuronal damage in the brain of hamsters offers a compelling explanation for the high incidence of hyposmia deficits reported in humans. The unresolved damage induced by XBB.1 and WH-09 highlights neurological threats from immune-evasive variants. BF.7 and BA.1 exhibited minimal neurostructural alterations in both primary and reinfection models. Syrian hamsters offer advantages for modeling neuropathogenesis induced by SARS-CoV-2 variants.

## MATERIALS AND METHODS

### Viruses and cell lines

The SARS-CoV-2 WH-09 strain (SARS-CoV-2/WH-09/human/2020/CHN; GenBank: MT093631.2), Omicron BA.1 variant (SARS-CoV-2/human/CHN/Omicron-1/2021; GenBank: OM095411.1), Omicron BF.7 variant (SARS-CoV-2/human/CHN/ ILAS-2023-1/2023; GenBank: OQ978950.1), and Omicron XBB.1 variant (SARS-CoV-2/human/CHN/ILAS/2023; GenBank: C_AA055151.1) utilized in this study were obtained from the Institute of Laboratory Animal Science (CAMS & PUMC, China).

All viruses were propagated in the Vero E6 cell line and subsequently titrated. Briefly, the virus was cultured in Vero E6 cells maintained in Dulbecco’s Modified Eagle Medium supplemented with 10% heat-inactivated fetal bovine serum and 1% penicillin-streptomycin. To determine the 50% tissue culture infective dose (TCID_50_), culture media containing the virus were serially diluted 10-fold and inoculated into Vero E6 cells. After a 3-day incubation period, the endpoint dilution was determined based on the presence of cytopathic effects (CPEs) in half of the replicate cultures. The Reed-Muench method was employed to calculate the viral titer.

### Animal experiment

The animals used in this study were 6- to 8-week-old specific-pathogen-free (SPF) Syrian hamsters, sourced from Beijing Vital River Laboratory Animal Technology Co., Ltd. The Syrian hamsters were intranasally inoculated with the four SARS-CoV-2 strain**s** (WH-09 strain, Omicron BA.1, BF.7, and XBB.1) at an equal dose of 1 × 10^5^ TCID_50_ per hamster. To evaluate differences in pathogenicity among these strains, Syrian hamsters were intranasally inoculated with WH-09, BA.1, BF.7, and XBB.1 (*n* = 6 per group, with equal numbers of males and females), and euthanized at 21 days post-infection (dpi). Furthermore, Syrian hamsters initially infected with the WH-09 strain were rechallenged with the four virus strains six months later. Euthanasia was performed at 21 days post-reinfection (dpr).

The heads with brains *in situ* within the cranium were extracted, post-fixed in 4% PFA for 24 h at 4°C, then transferred to PBS with 0.01% sodium azide. Before *ex vivo* MRI imaging, the head of the hamster was rehydrated by immersion in a 1:150 solution of Prohance/PBS for 72 h.

### *Ex vivo* MRI acquisition

All neuroimaging was performed on a 7.0 T animal MRI system (Agilent Technologies Inc., Palo Alto, CA, USA) with a 20-mm volume coil. Prior to MRI, brains were immersed in susceptibility-matching perfluoropolyether (Fomblin) to eliminate air-tissue interfaces. Eight healthy hamsters (6–8 weeks old) were used for constructing the MRI template.

3D T2-weighted isotropic images were acquired with a Fast Spin-Echo sequence with the following parameters: (TR/TE = 1,000/13 ms; Matrix = 256 × 192×192; field of view (FOV) = 25.6 × 19.2 × 19.2 mm^3^; Resolution = 100 × 100 × 100 µm^3^; 1 averages; Scan time =1 h:16 min:50 s).

3D T2*-weighted isotropic images were acquired with a Gradient-Echo (GRE) sequence with the following parameters: TR/TE = 6/3 ms; Matrix=256 × 192×192; FOV = 25.6 × 19.2 × 19.2 mm^3^; Resolution = 100 × 100 × 100 µm^3^; 25 averages; Scan time =1 h:30 min:45 s.

DTI images were acquired with spin echo diffusion preparation: TR/TE = 1,500/30 ms; 30 diffusion directions; b-value = 1,000 s/mm²; Matrix = 128 × 128; FOV = 25 × 25 mm^2^; Resolution=195 × 195 µm^2^; 4 averages; Scan time = 31 min:32 s.

### MRI data processing & analysis

Post-processing of T2-weighted MRI structural images was performed using ANTs (Advanced Normalization Tools). First, non-brain tissues were manually removed from the images. Subsequently, intensity non-uniformity correction was applied using the N3 method. A representative hamster T2*-weighted MRI structural image (exhibiting bilateral symmetry in the coronal plane) was selected as the initial template. The T2*-weighted structural images from the remaining seven hamsters were registered to this template space using a two-stage approach: initial rigid registration followed by diffeomorphic nonlinear registration. Following registration, the initial template and the seven registered images were arithmetically averaged to generate a new template. All eight images were then registered to this new template space using the aforementioned registration method. Finally, the eight registered data sets were arithmetically averaged to produce the final template image. Anatomical labeling of hamster brain structures was performed on this final averaged template, referencing the histochemical brain atlas of Syrian hamsters.

Volumetric analysis of olfactory bulb, hippocampus, piriform cortex, and entorhinal cortex using manual segmentation (ITK-SNAP) on T2 and T2*, respectively. T2 signal intensity of the region of interest (ROI) was also calculated.

Preprocessing (eddy current and motion correction, tensor fitting) using DSI Studio. Mean values of DTI metrics (FA, MD, axial diffusivity, radial diffusivity) were extracted from anatomically defined ROIs (olfactory bulb, corpus callosum, hippocampus, piriform cortex, entorhinal cortex) delineated on the template or individual space after registration. ROIs were defined using a digital atlas or manual tracing. A two-tailed unpaired t-test was used to compare the control and infection groups. *P* < 0.05 was considered statistically significant.

### H&E study

Coronal sections of formalin-fixed hamster brains (5 μm thickness) were prepared using a rotary microtome, with samples containing cerebral cortex and hippocampus regions selected for analysis. Following dehydration through an ethanol gradient, xylene clearing, and paraffin embedding, serial sections were stained with H&E. The cortex and hippocampal structures were examined under a light microscope (Zeiss, Primo star), with images captured using an integrated digital imaging system.

## SUPPLEMENTAL MATERIAL

Supplemental Material spectrum.03567-25-s0001.docx Figures S1 to S4 and Table S1.

ASM does not own the copyrights to Supplemental Material that may be linked to, or accessed through, an article. The authors have granted ASM a non-exclusive, world-wide license to publish the Supplemental Material files. Please contact the corresponding author directly for reuse.
