# Supplementary material for: MRI of Syrian hamsters reveals SARS-CoV-2 variant-specific neuroinflammation
Source: Microbiol Spectr. 2026 Feb 3;14(3):e03567-25. doi: 10.1128/spectrum.03567-25 (PMC12955404; doi:10.1128/spectrum.03567-25)
Supplement: Supplemental Material — Figures S1 to S4 and Table S1. [file spectrum.03567-25-s0001.docx]

**Supplemental Information**

**MRI of Syrian Hamsters Reveals SARS-CoV-2 Variant- Specific Neuroinflammation**

Yisi Tang ^a,1^, Xiaohui Wei ^a,1^, Kai Gao ^a^, Bowen Guan ^a^,Jiangning Liu ^a,^ *, Xudong Shi ^a,^ *

^a^ *Institute of Laboratory Animal Science, CAMS & PUMC, National Human Diseases Animal Model Resource Center,* *National Center of Technology Innovation for Animal Model, State Key Laboratory of Respiratory Health and Multimorbidity, NHC Key Laboratory of Human Disease Comparative Medicine, Beijing 100021, China*

^1^ These authors contributed equally to this work.

*Corresponding author: [liujn@cnilas.org](mailto:liujn@cnilas.org), [shixudong@cnilas.org](mailto:shixudong@cnilas.org)


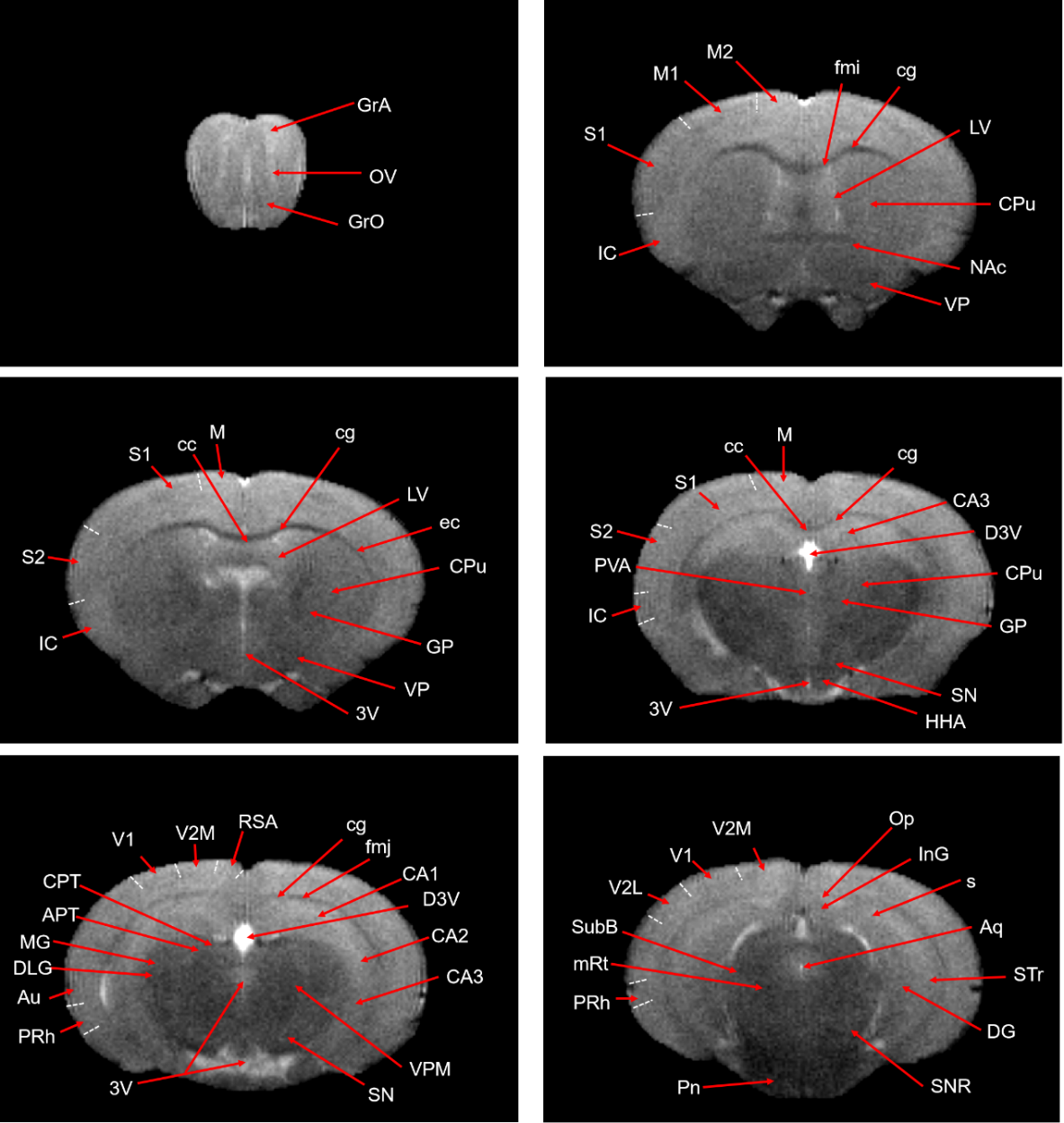


**Figure S1.** Six representative T2-weighted coronal images with structural annotations. (abbreviations correspond to Table S1)

**Table S1.** The abbreviations of regions of hamster brain

| Abbr. | Brain regions |
| --- | --- |
| Aq | aqueduct |
| Au | primary auditory cortex |
| CA3 | field CA3 of hippocampus |
| cg | cingulum |
| CPu | caudate putamen(striatum) |
| DG | dentate gyrus |
| DLG | dorsal lateral geniculate nu |
| DM | dorsomedial hypothalamic nuclear |
| D3V | 3rd ventricle |
| ec | external capsule |
| fmi | forceps minor corpus callosum |
| fmj | forceps major corpus callosum |
| GP | globus pallidus |
| GrA | granule cell layer of the accessory olfactory bulb |
| GrO | granule cell layer of the olfactory bulb |
| HHA | hypothalamus |
| IC | insular cortex |
| LH | lateral hypothalamic |
| InG | intermediate gray of sup coll |
| LV | lateral ventricle |
| M1 | primary motor cortex |
| M2 | secondary motor cortex |
| MG | medial geniculate nu |
| mRt | mesencephalic reticular formation |
| Nac | nucleus accumbens |
| Op | optic layers of superior coll |
| OV | olfactory ventricle |
| PH | posterior hypothalamic areas |
| Pn | pontine nuclei |
| Po | posterior thalamic nuclear group |
| PRh | perirhinal cortex |
| PVA | paraventricular thalamus anterior |
| Re | reuniens thalamic nu |
| RSA | agranular retrosplenial cortex |
| s | subiculum |
| S1 | primary somatosensory cortex |
| S2 | secondary somatosensory cortex |
| SN | substantia nigra |
| SNR | substantia nigra reticular part |
| STr | subiculum transition area |
| SubB | subbrachial nucleus |
| V1 | primary visual cortexc |
| V2L | secondary visual cortex lateral |
| V2M | secondary visual cortex medial |
| VP | ventral pallidum |
| VPL | ventral posterolateral thalamic nuclear |
| VPM | ventral posteromedial thalamic nuclear |
| ZI | zona incerta |
| 3V | 3rd ventricle |


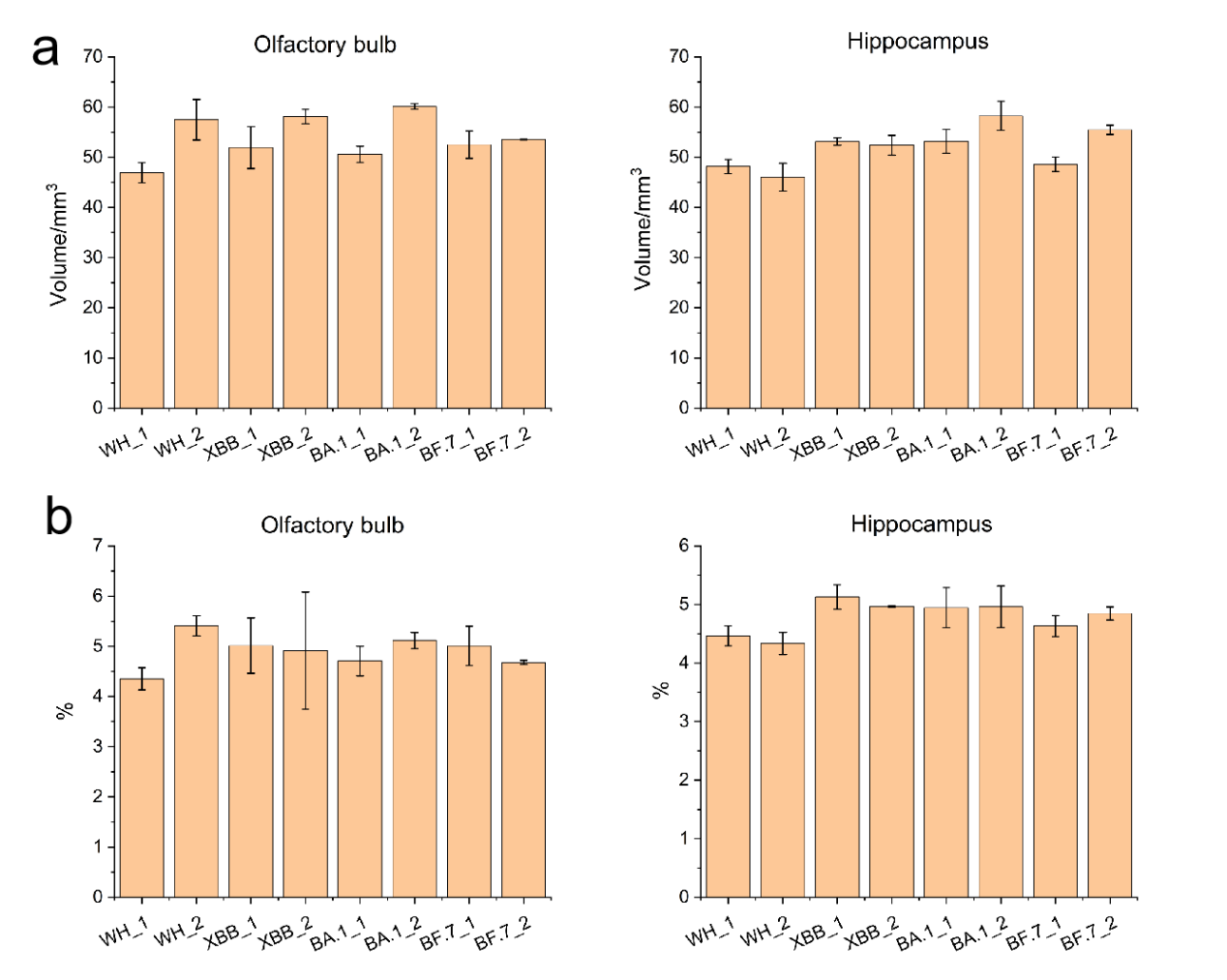


**Figure S2.** (a) The volume of olfactory bulbs and hippocampus in hamster with primary infection and reinfection by different SARS-CoV-2 strains. (b) The ratio of olfactory bulbs and hippocampus in the whole brain of hamsters


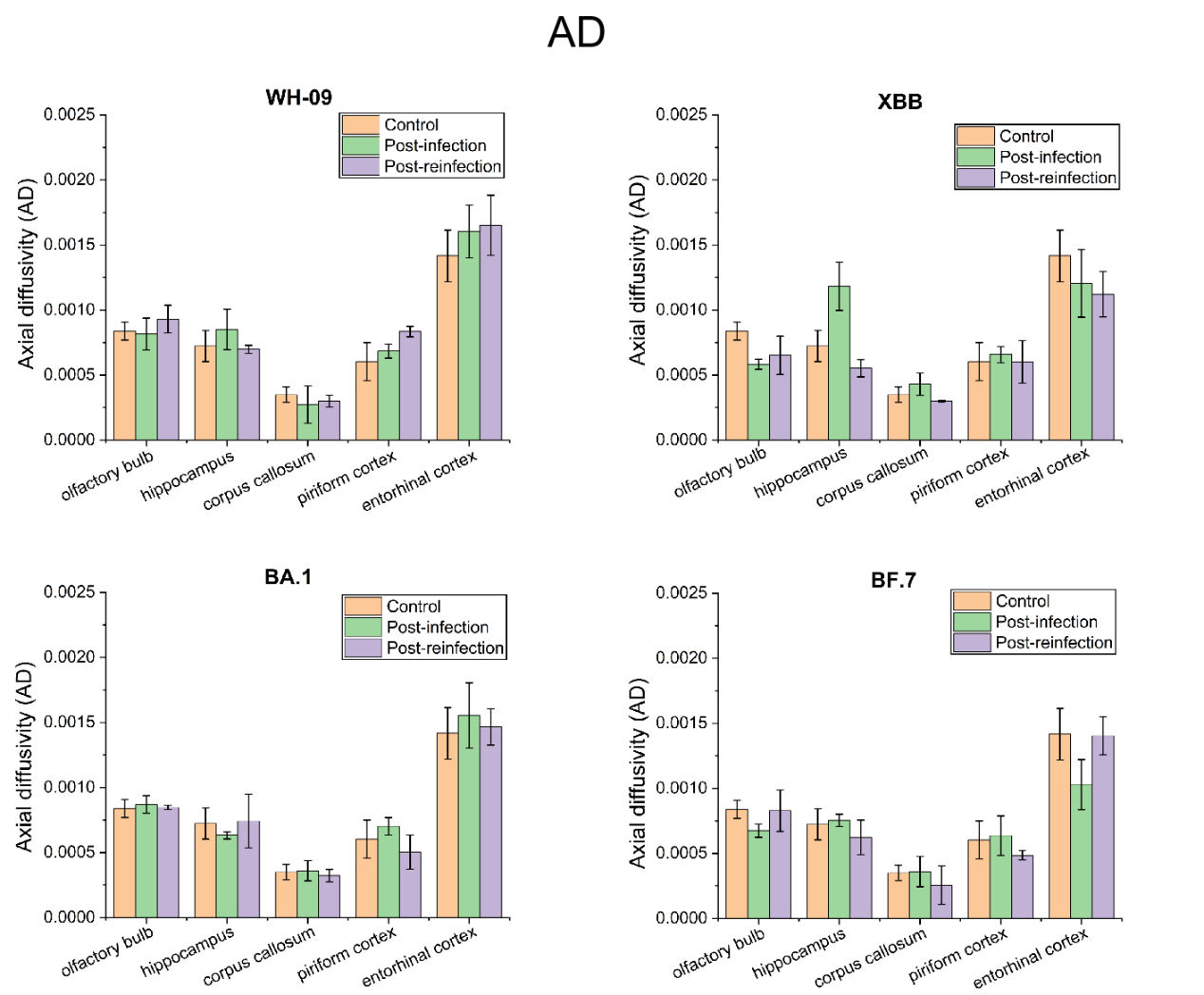


**Figure S3.** Axial diffusivity (AD) of brain regions (olfactory bulb, hippocampus, corpus callosum, piriform cortex, entorhinal cortex) in hamster with primary infection and reinfection by different SARS-CoV-2 strains.


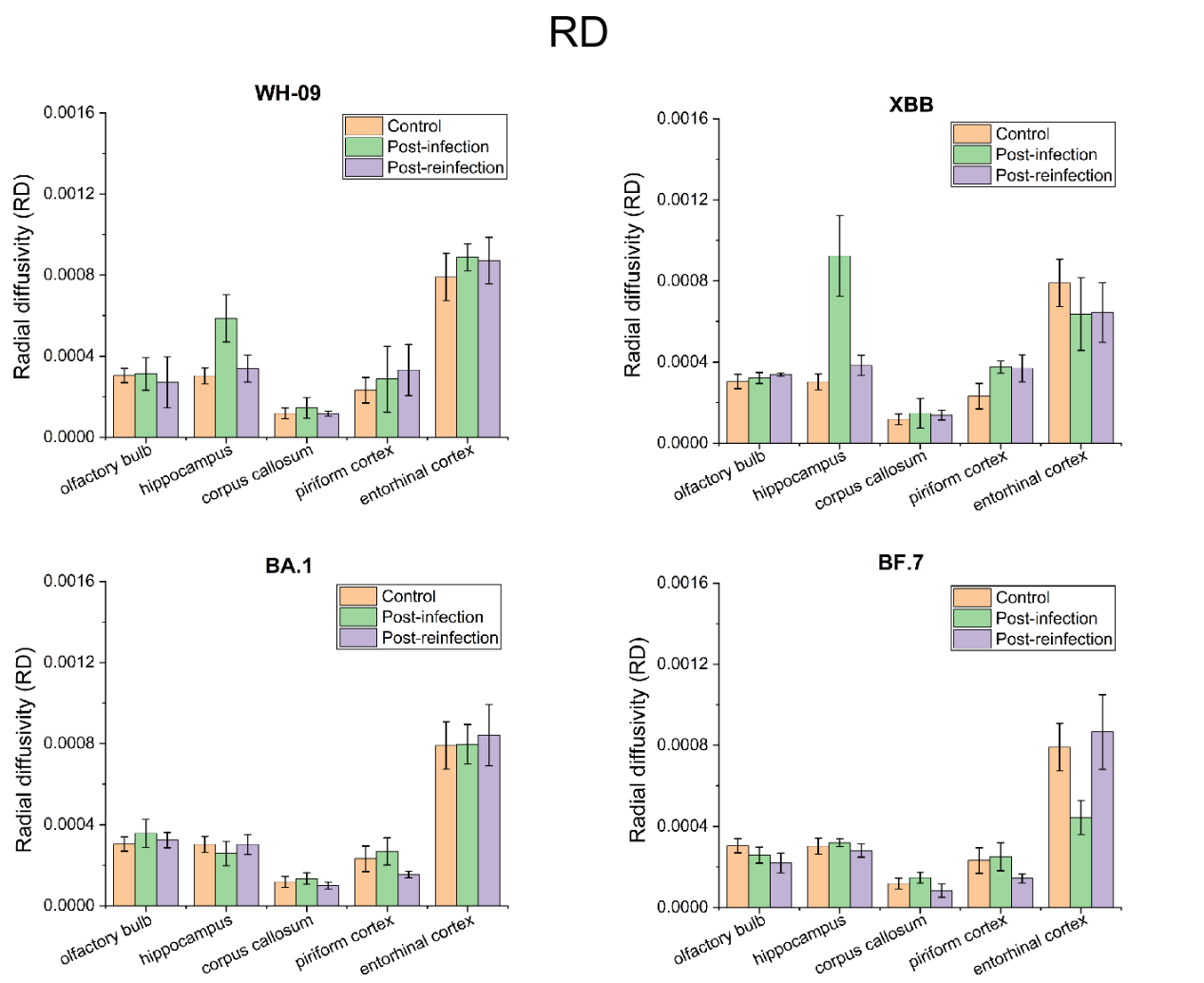


**Figure S4.** Radial diffusivity (RD) of brain regions (olfactory bulb, hippocampus, corpus callosum, piriform cortex, entorhinal cortex) in hamster with primary infection and reinfection by different SARS-CoV-2 strains.
